# New Hydrophilic Helixone Dialyzers as an Alternative for Hemodialysis Patients With Membrane Hypersensitivity Reactions: A Retrospective Single‐Cohort Study

**DOI:** 10.1111/hdi.70056

**Published:** 2026-02-01

**Authors:** Víctor Joaquín Escudero‐Saiz, Elena Cuadrado‐Payán, Eugenia Castellote, Roxana Buri, Higini Cao, Silvia Collado, Lida Maria Rodas, Néstor Fontseré, José Jesús Broseta, Francisco Maduell

**Affiliations:** ^1^ Nephrology and Renal Transplantation Department Hospital Clínic of Barcelona Barcelona Barcelona Spain; ^2^ Nephrology Department Consorci Hospitalari de Vic Vic Barcelona Spain; ^3^ Nephrology Department Hospital Figueres Figueres Girona Spain; ^4^ Nephrology Department Hospital del Mar Barcelona Barcelona Spain; ^5^ Department of Medicine University of Barcelona Barcelona Barcelona Spain

**Keywords:** cellulose triacetate, hydrophilic helixone, hypersensitivity reactions, synthetic dialysis membranes

## Abstract

**Background:**

Synthetic dialysis membranes, particularly those composed of polysulfone blended with polyvinylpyrrolidone, are commonly used in hemodialysis due to their efficiency. However, hypersensitivity reactions—often atypical and not fitting traditional type A and B classifications—have increasingly been reported. Symptoms include chest tightness, bronchospasm, oxygen desaturation, and hypotension, often without clear etiology. The need for safer alternatives has led to interest in newer dialyzer technologies, such as the hydrophilic helixone membrane which incorporates tocopherol to enhance biocompatibility.

**Methods:**

We conducted a multicenter, retrospective observational study in four hemodialysis units in Catalonia (Spain). Thirty‐one stable patients previously diagnosed with hypersensitivity to synthetic membranes and dialyzing with cellulose triacetate were switched to hydrophilic helixone (CorAL) dialyzers. Clinical data, including symptoms before and after the switch, were extracted from medical records. The primary outcome was the occurrence of adverse reactions after the transition.

**Results:**

Of the 31 patients (mean age 70.1 ± 13.9 years), the most common symptoms at initial reaction included cutaneous (21, 67.7%), respiratory (13, 41.9%), and cardiovascular (9, 29.0%) manifestations. After switching to hydrophilic helixone, 28 patients (90.3%) had no further hypersensitivity symptoms over a mean follow‐up of 5.8 ± 4.3 months. Three patients experienced mild reactions (two with pruritus, one with hypotension) and reverted to cellulose triacetate. No significant associations were found between relapse and the type of membrane or symptom profile.

**Conclusions:**

The hydrophilic helixone membranes appear to be a safe and well‐tolerated alternative for patients with a history of hypersensitivity to synthetic membranes. These findings suggest that hydrophilic helixone dialyzers may allow more patients to continue treatment with synthetic membranes without severe reactions, potentially improving biocompatibility and treatment flexibility in routine clinical practice. While the findings are promising, larger prospective studies are necessary to confirm the safety and long‐term clinical benefits.

## 1 Introduction

The number of patients requiring renal replacement therapy continues to increase, highlighting the urgent need for safe and well‐tolerated dialysis options [1, 2, 3]. Synthetic membranes of the polyarylsulfonate family (polysulfone or polyethersulfone) bent with polyvinylpyrrolidone (PVP) are widely used in hemodialysis treatments due to their high permeability and efficiency [4]. However, there is a growing concern regarding the relatively frequent occurrence of hypersensitivity reactions, which can lead to adverse intradialytic events [5].

Hypersensitivity reactions were traditionally classified into two categories: type A reactions are typically acute, IgE‐mediated, and present within minutes with symptoms such as hypotension, dyspnea, or chest tightness, whereas type B reactions are usually delayed, less severe, and often manifest as nonspecific symptoms such as pruritus or mild respiratory complaints [6, 7]. There are a variety of potential triggers in dialysis treatments, such as common medications, the interaction with AN69 polyacrylonitrile membranes and angiotensin‐converting‐enzyme inhibitors [8], and other materials used in dialyzers and tubing, especially concerning their sterilization methods (like ethylene oxide sterilization, which was used in the past), have all been identified as possible contributors to these reactions [9, 10].

However, in the twenty‐first century, a new type of reaction to synthetic dialyzers has emerged—one that does not fully meet the criteria for classic type A reactions. They are generally more common but less severe than type A reactions and, in some cases, resemble severe type B ones. These reactions present variable onset times, ranging from 5 to 180 min after the initiation of dialysis. The most frequently reported symptoms include chest tightness, hypoventilation or bronchospasm, oxygen desaturation, and hypotension. Although these reactions have been associated with synthetic dialyzers—given that they do not typically occur with cellulose triacetate membranes—the exact cause remains unknown. Potential contributing factors include the presence of PVP, sterilization methods, storage conditions, as well as possibly other unidentified factors [11, 12, 13, 14, 15]. The incidence of these reactions has been documented at approximately 2% [10]. The role of nursing is essential in the detection, initial management, and prevention of hypersensitivity reactions to dialyzers [16].

Moreover, an association between synthetic membranes and the presence and severity of pruritus has been reported. One clinical study found that patients dialyzed with polysulfone membranes experienced pruritus more frequently than those treated with hemophane or cuprophane membranes, implying a potential role of membrane biocompatibility. Less biocompatible membranes may trigger greater immune activation and the release of inflammatory mediators, contributing to symptoms [17].

Thus, there is a need for alternative dialyzer materials to enhance patient safety and improve treatment efficacy. Cellulose triacetate or polymethylmethacrylate (PMMA) based membranes are an alternative option for avoiding these clinical complications [18]. A promising new development is the hydrophilic helixone membrane, which combines a blend of polysulfone and PVP, with small amounts of tocopherol added to stabilize the blood‐side surface [19]. This modification promotes the formation of a more robust hydrophilic layer, which may help reduce oxidative stress and immune activation factors often associated with hypersensitivity reactions [20, 21]. In vitro studies have demonstrated that this new membrane results in lower complement activation (C3a, C5a, and C5b‐9) [19], reduced platelet loss [22], and increased hydrophilicity when compared to other polysulfone membranes [23]. These findings have also been supported by clinical studies, which reported lower levels of complement, leukocyte, and platelet activation in patients undergoing hemodiafiltration with these membranes [20, 24].

The primary aim of this study was to evaluate the safety and tolerability of hydrophilic helixone dialyzers in patients with a history of hypersensitivity reactions to synthetic membranes, who switched from cellulose triacetate membranes to the hydrophilic helixone dialyzer based on clinical judgment, a highly selected, high‐risk population, for whom re‐exposure to polysulfone‐derived membranes would not normally be considered. The secondary objectives were to characterize the clinical presentation of prior hypersensitivity episodes and to assess the association between symptom profiles and subsequent reactions after switching dialyzer membranes.

## 2 Materials and Methods

This was a retrospective, observational, multicenter, single‐cohort study conducted in four hemodialysis units in Catalonia, Spain: Hospital Clínic Barcelona, Consorci Hospitalari de Vic, Hospital del Mar, and Hospital de Figueres. The data collection was performed in the second quarter of 2025.

All prevalent patients undergoing chronic hemodialysis in these centers were screened to identify those who had experienced a clinically diagnosed hypersensitivity reaction to synthetic dialysis membranes, as determined by the nephrologist in charge. Patients who were subsequently treated with cellulose triacetate membranes and later switched to hydrophilic helixone membranes (CorAL dialyzer series, Fresenius Medical Care, Bad Homburg, Germany) by the treating physician were included in the analysis. No patients meeting these criteria were excluded.

Dialysis prescriptions typically consisted of three sessions per week, each lasting 4–5 h, with blood flow rates of 300–450 mL/min and dialysate flow rates of 400–700 mL/min, according to the routine clinical practices at each center. These parameters remained unchanged during the study period and no other changes were made to the patient's medication besides the membrane exchange. The decision to switch to the hydrophilic helixone dialyzer was made individually by each nephrologist, based on membrane availability and clinical judgment; no systematic protocol was applied.

Data were extracted retrospectively from electronic medical records. The variables collected included patients' age, sex, etiology of chronic kidney disease, type of vascular access, anticoagulation strategy, dialysis vintage, and the time from dialysis initiation to the first hypersensitivity reaction. Additionally, information was recorded on the dialyzer used at the time of the initial reaction as well as the detailed symptomatology of both the debut and any relapse reactions.

The primary outcome was the occurrence of hypersensitivity reactions after switching to the hydrophilic helixone dialyzer. Secondary outcomes included the characterization of initial reaction profiles and the description of any relapse events.

The results are expressed as the arithmetic mean ± standard deviation. Fisher's exact test was used for the analyses between qualitative or ordinal variables as *n* < 5 in the variable of study (relapses) and *U*‐Mann Whitney for quantitative ones. *p*‐values < 0.05 were considered statistically significant. Analyses were performed using SPSS software version 23 (IBM SPSS Statistics, Chicago, IL, USA).

## 3 Results

**FIGURE 1:**
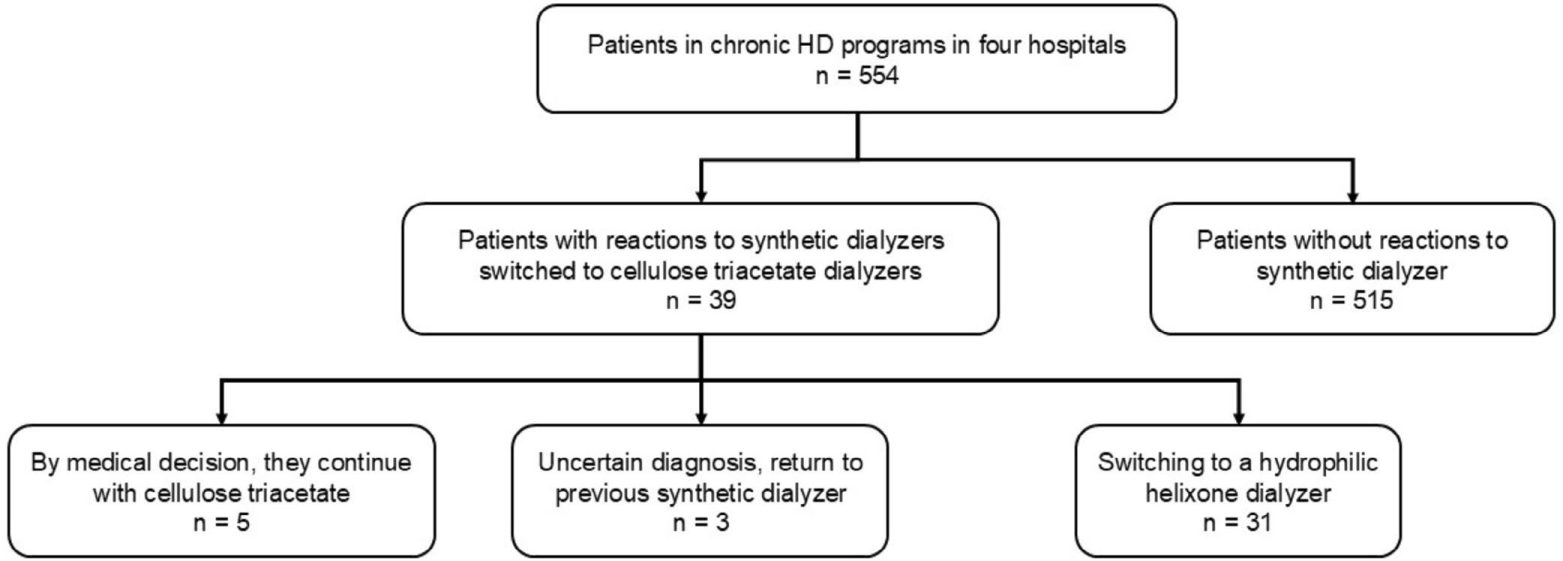
Flow chart of included patients.

From a total of 554 prevalent patients from the four hemodialysis units, 31 patients were included (36% women), with a mean age of 70.1 ± 13.9 years (range 41–96), who were stable in a thrice‐weekly hemodialysis program (Figure 1). Remarkably, five patients maintained cellulose triacetate per clinical decision, and three cases were not considered, as the diagnosis of synthetic dialyzer hypersensitivity reaction was uncertain.

The baseline characteristics of the patients are summarized in Table 1. On average, patients had been stable on dialysis for 40.3 ± 33.7 months before experiencing their first hypersensitivity reaction to synthetic dialysis membranes. Notably, nine patients (29%) developed this reaction during their first month of hemodialysis. The most common type of membrane associated with hypersensitivity reactions was the earlier helixone generation, whereas five patients were using polyethersulfone dialyzers (Table 1).

**TABLE 1:** Demographic characteristics from patients.

Variable	Mean ± standard deviation/*N* (%)	
Age (years)	70.1 ± 13.9	
Women	11 (35.5%)	
Chronic kidney disease etiology	Diabetic nephropathy: 12 (38.7%)	
	Hypertensive nephrosclerosis and renovascular disease: 8 (25.8%)	
	Unknown: 5 (16.1%)	
	Chronic glomerulonephritis: 3 (9.7%)	
	Polycystic kidney disease: 2 (6.5%)	
	Systemic diseases: 1 (3.2%)	
Vascular access	Native arterio‐venous fistula: 14 (45.2%)	
	Prosthetic arterio‐venous fistula: 1 (3.2%)	
	Tunneled central venous catheter: 16 (51.6%)	
Anticoagulation strategy	Low molecular weight heparin: 16 (51.6%)	
	Non‐fractioned heparin: 11 (35.5%)	
	None: 4 (12.9%)	
Vintage time (months on HD prior to hypersensitivity reaction)	40.3 ± 33.7	
Time from start of hemodialysis to onset of hypersensitivity reaction (days)	426 ± 639 (range 1–2877)	
Debut reaction dialyzer	FX CorDiax 60	19 (61.3%)
	FX CorDiax 80	5 (16.1%)
	Revaclear 400	2 (6.5%)
	FX CorDiax 100	1 (3.2%)
	F‐10 HPS	1 (3.2%)
	Phylther HF 17G	1 (3.2%)
	Theranova 400	1 (3.2%)
	BLS819SD	1 (3.2%)

**FIGURE 2:**
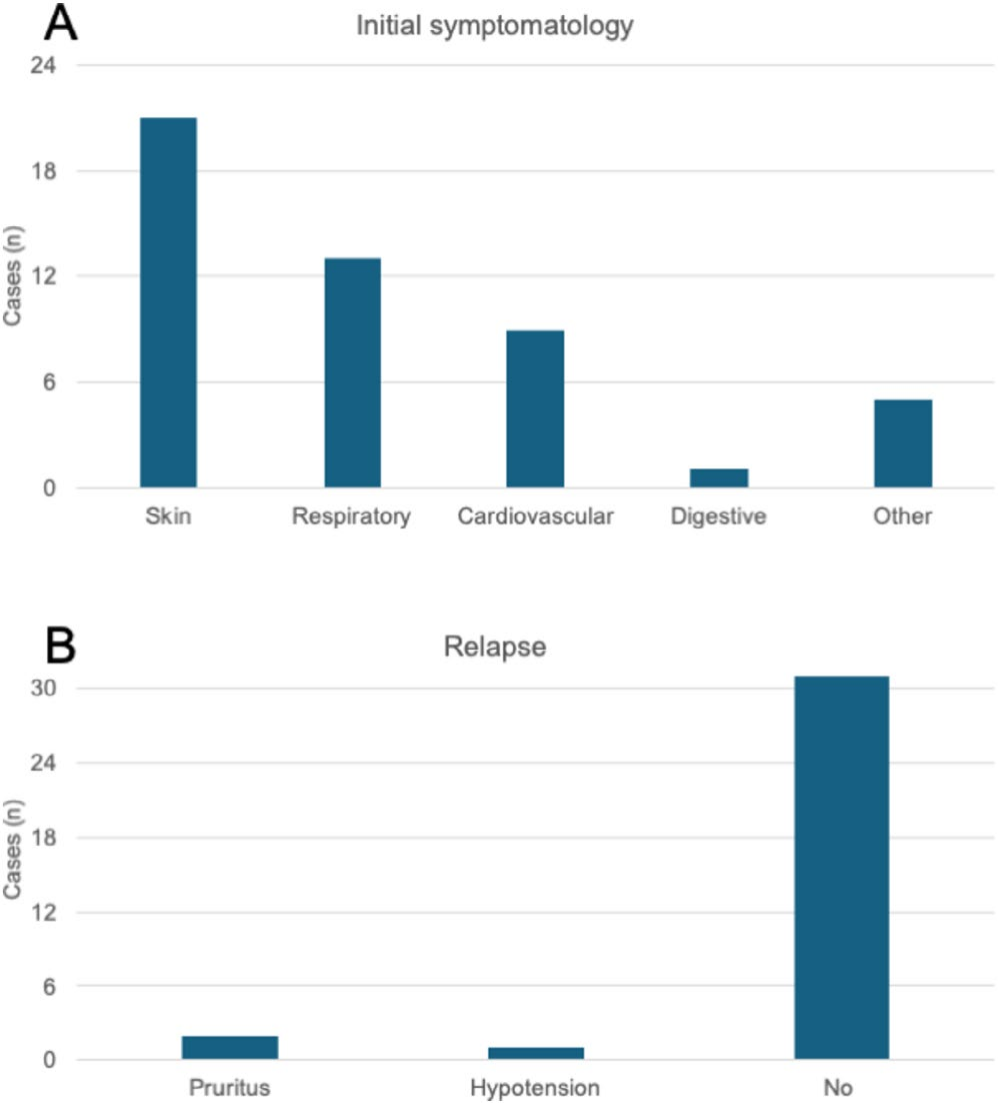
Symptomatology of patients in the incompatibility reaction diagnosis (a) and relapse (b).

The symptoms experienced by patients during the hypersensitivity reaction are detailed individually in Table 2. The most commonly affected system was the skin (67.7%), followed by the respiratory (41.9%) and the cardiovascular (29.0%) systems. The digestive system was the least affected, with only one case (Figure 2). The majority of patients experienced symptoms in one system (21 patients, 67.7%), followed by two systems (six patients, 19.4%) and three systems (four patients, 12.95%). After the clinical suspicious diagnosis of synthetic dialyzers hypersensitivity reaction, patients were changed to cellulose triacetate membranes. The duration from the change to cellulose triacetate to the decision to switch to the hydrophilic helixone dialyzer was 644 ± 657 days (range 1–2710).

**TABLE 2:** Initial symptomatology of incompatibility synthetic dialysis membrane reaction of each patient.

N	Sex	Age	Membrane	Symptomatology
1	Female	91	Helixone (FX CorDiax 60)	Pruritus
2	Male	78	Helixone (FX CorDiax 60)	Pruritus, erythema on chess and oxygen desaturation
3	Female	96	Polietersulfone (Theranova 400)	Pruritus and erythema on face
4	Male	70	Helixone (FX CorDiax 60)	Pruritus and erythema on neck and chess
5	Male	68	Polietersulfone (Phylter HF 17G)	Erythema on chess and arms with oxygen desaturation
6	Male	81	Polietersulfone (Revaclear 400)	Pruritus
7	Male	73	Helixone (FX CorDiax 60)	Hypotension without other possible etiology
8	Male	75	Polietersulfone (BLS819SD)	Erythema on face
9	Male	49	Helixone (FX CorDiax 60)	Pruritus and erythema on neck and face
10	Female	82	Helixone (FX CorDiax 60)	Face erythema
11	Male	87	Helixone (FX CorDiax 60)	Pruritus
12	Male	78	Polietersulfone (Revaclear 400)	Face erythema, oxygen desaturation, and hypotension
13	Female	41	Helixone (FX CorDiax 60)	Pruritus
14	Female	62	Helixone (FX CorDiax 60)	Pruritus
15	Female	78	Helixone (FX CorDiax 60)	Oxygen desaturation and hypotension
16	Female	50	Helixone (FX CorDiax 60)	Dyspnea without oxygen desaturation
17	Male	60	Helixone (FX CorDiax 60)	Pruritus
18	Male	80	Helixone (FX CorDiax 80)	Pruritus
19	Female	78	Polietersulfone (F‐10HPS)	Pruritus
20	Male	81	Helixone (FX CorDiax 60)	Pruritus and face erythema.
21	Male	49	Helixone (FX CorDiax 60)	Pruritus
22	Male	64	Helixone (FX CorDiax 80)	Pruritus
23	Female	59	Helixone (FX CorDiax 60)	Pruritus
24	Male	50	Helixone (FX CorDiax 60)	Erythema with oxygen desaturation and hypotension
25	Male	65	Helixone (FX CorDiax 60)	Pruritus, erythema and oxygen desaturation with hypotension and atypical chest pain
26	Male	76	Helixone (FX CorDiax 80)	Oxygen desaturation with hypotension and upper limbs tremor
27	Male	69	Helixone (FX CorDiax 80)	Oxygen desaturation with hypotension and upper limbs tremor
28	Male	74	Helixone (FX CorDiax 60)	Oxygen desaturation without other etiology
29	Male	69	Helixone (FX CorDiax 80)	Oxygen desaturation, hypotension and abdominal pain with headache
30	Female	89	Helixone (FX CorDiax 60)	Oxygen desaturation, hypotension and atypical chest pain
31	Female	52	Helixone (FX CorDiax 100)	Oxygen desaturation without other etiology

After the introduction of hydrophilic helixone membranes, there were three (9.7%) clinical relapses recorded. Two patients experienced pruritus again, while one patient exhibited hypotension, occurring 1 h earlier than in previous treatments. The three patients who experienced relapses were on the hydrophilic helixone membrane for a median duration of 22 days (range 16–34) and were subsequently switched back to cellulose triacetate membranes. In contrast, 28 patients (90.3%) continued using the new hydrophilic helixone dialyzers, with a median follow‐up period after the introduction of the hydrophilic helixone of 5.8 ± 4.3 months (range 0.2–15).

Fisher's exact test did not show any statistically significant relationship between relapses and the type of clinical system affected or the type of synthetic membrane used (*p*‐value 0.728 and *p*‐value 0.422, respectively).

## 4 Discussion

Polysulfone hypersensitivity has been widely documented as a cause of adverse events during hemodialysis, including cutaneous, respiratory, and hemodynamic manifestations [5]. In these cases, cellulose triacetate or PMMA membranes have been widely used, as they represent a safe alternative for sustaining hemodialysis treatment [18]. This study presents, to our knowledge, the first clinical evidence of the potential use of new hydrophilic helixone membranes as an additional alternative to the mentioned dialyzers for patients who have experienced synthetic dialyzer hypersensitivity reactions, resulting in few and non‐severe clinical relapses.

After the change from cellulose triacetate to hydrophilic helixone, three patients presented symptomatology attributable to a relapse. Two patients presented pruritus, which is an unspecific and challenging symptom for the clinician, as it requires ruling out other causes (such as cutaneous diseases, infections, or other systemic etiologies with cutaneous manifestations) [25] before attributing it to a synthetic dialyzer hypersensitivity reaction; in fact, uremic pruritus may be challenging to dismiss in the diagnosis process as there are no specific biomarkers for it neither for hypersensitivity synthetic membrane reaction. Besides, the other patient presented hypotension, which is a frequent complication during hemodialysis, influenced by several factors, including ultrafiltration rate, dialysate composition, patient comorbidities, and endothelial dysfunction [26].

Hemodialysis techniques and materials have evolved since their beginning. The first documented intradialytic anaphylactoid reactions (type A) were IgE‐mediated and related to previous sterilizing agents, such as ethylene oxide [6, 27], and also the concurrence of AN69 polyacrylonitrile membranes and angiotensin‐converting‐enzyme inhibitor treatment, which increased the upper regulation of the bradykinin pathway [8]. The avoidance of those materials led to pseudo‐anaphylactic reactions being attributed to the membrane's degree of biocompatibility. Recently, it has been demonstrated that basophil degranulation is activated during incompatibility reactions [28] among other factors [29]. This membrane's biocompatibility is related to the degree of activation of the immune system [30], platelet and protein adsorption [31, 32] and complement activation [33], summarizing a more complex relationship between the interaction of blood with the membrane material [29]. Hypersensitivity reactions to synthetic membranes can occur even in the absence of classic risk factors, with a potentially higher risk in the elderly due to immunosenescence [28].

The way hydrophilic helixone might have inhibited hypersensitivity reactions could be linked to tocopherol's antioxidant properties, which mitigate oxidative stress [21]. In laboratory studies, significant variations in PVP content and elution, as well as differences in the formation of secondary membranes, were observed among various dialyzer membranes [19, 34, 35, 36]. These changes contribute to the development of a sturdier hydrophilic layer, which enhances the hemocompatibility of polysulfone membranes by improving their antifouling capabilities, leading to reduced protein adsorption and decreased coagulation activation [19, 22, 34, 35, 36].

It is described that this type of membrane reduces protein and platelet adhesion to the membrane, as well as complement activation in vitro. Furthermore, some recent trials have yielded lower leukocyte and complement activation during dialysis with hydrophilic helixone membranes [20, 37]. Ehlerding et al. assessed in vivo the biocompatibility profile and efficacy of FX CorAL 600 compared to other conventional membranes based on polyarylethersulfone or cellulose triacetate. They observed lower C3a and C5a activation during the first 15 min of therapy and lower sC5b‐9 activation during the entire hemodialysis session with hydrophilic helixone membranes compared to these other membranes [37]. Further studies from the same study group reported a lower leukocyte and platelet drop after initiation, as well as the lowest levels of leukocyte and platelet activation markers (LTB‐4 and PMN elastase, respectively) and β‐TG [20]. Together, they may play a key role in immune activation and reduce the so‐called hypersensitivity reactions observed in our cohort.

The efficacy of these new hydrophilic dialyzers has already been published in a previous study, showing that the new generation hydrophilic helixone dialyzers series maintain the effectiveness and albumin loss achieved by the previous most advanced helixone generation [38].

By demonstrating a high tolerance to hydrophilic helixone dialyzers in patients previously intolerant to synthetic membranes, this study highlights the importance of incorporating new biocompatible technologies into standard care pathways to enhance safety and flexibility in dialysis treatment. Despite these promising results, this study has several limitations. First, the cohort was relatively small and included only patients from four dialysis units in Catalonia, which may limit the generalizability of the findings to other settings or populations. Second, the mean follow‐up period was less than 6 months, restricting the ability to assess long‐term safety and effectiveness. Third, as a retrospective observational single‐cohort study, there is potential selection bias because some patients remained on cellulose triacetate membranes instead of switching to hydrophilic helixone based on clinical judgment—possibly due to more severe or recurrent hypersensitivity reactions—which could underestimate the true relapse rate. Additionally, the data collection relied on electronic health records and routine clinical documentation rather than standardized research instruments, which may have introduced variability in the classification of reactions and other variables. Fourth, the lack of data on dialysis adequacy, phosphate levels, and parathyroid hormone may influence how we interpret symptoms attributed to hypersensitivity reactions; however, a thorough medical history and physical examination can usually distinguish between chronic uremic or phosphoric pruritus and an allergic reaction. Finally, although all reactions were diagnosed by nephrologists or trained nursing staff, the lack of objective confirmatory testing limits the reproducibility of the diagnostic process. Future larger prospective studies and randomized clinical trials will be essential to validate these preliminary observations and clarify their applicability in diverse clinical contexts.

In conclusion, our findings indicate that the new hydrophilic helixone dialyzer series may represent a promising alternative to cellulose triacetate or PMMA membranes for patients who have experienced hypersensitivity reactions to synthetic membranes, potentially improving biocompatibility, treatment flexibility, and patient comfort in routine clinical practice. However, these results should be interpreted with caution due to the limited sample size, short follow‐up period, and the retrospective design of the study, which may restrict generalizability. Future larger prospective studies and randomized clinical trials are necessary to confirm these observations and to better define the long‐term safety, effectiveness, and impact on multidisciplinary management in hemodialysis.

## Funding

The authors have nothing to report.

## Ethics Statement

The study was conducted in accordance with the Declaration of Helsinki and approved by the local Ethics Committee of Hospital Clínic Barcelona (protocol code HCB/2018/1168). Patient consent was waived due to the retrospective nature of this study. However, the patients did sign a patient consent form for the use of their health data for research purposes, “Research in chronic renal failure. Review and observational studies.”

## Conflicts of Interest

F.M. has received consultancy and lecture fees from Fresenius Medical Care, Nipro, Toray, and Vifor. J.J.B. has received lecture fees from Fresenius Medical Care. The other authors declare no conflicts of interest.

## Data Availability

The data that support the findings of this study are available from the corresponding author upon reasonable request.
